# Supplementary material for: Stitching and registering highly multiplexed whole-slide images of tissues and tumors using ASHLAR
Source: Bioinformatics. 2022 Aug 16;38(19):4613–21. doi: 10.1093/bioinformatics/btac544 (PMC9525007; doi:10.1093/bioinformatics/btac544)
Supplement: btac544_Supplementary_Data [file btac544_supplementary_data.pdf]

Figure S1: Registration accuracy comparison on t-CyCIF human colon

A Registration error kernel density estimate

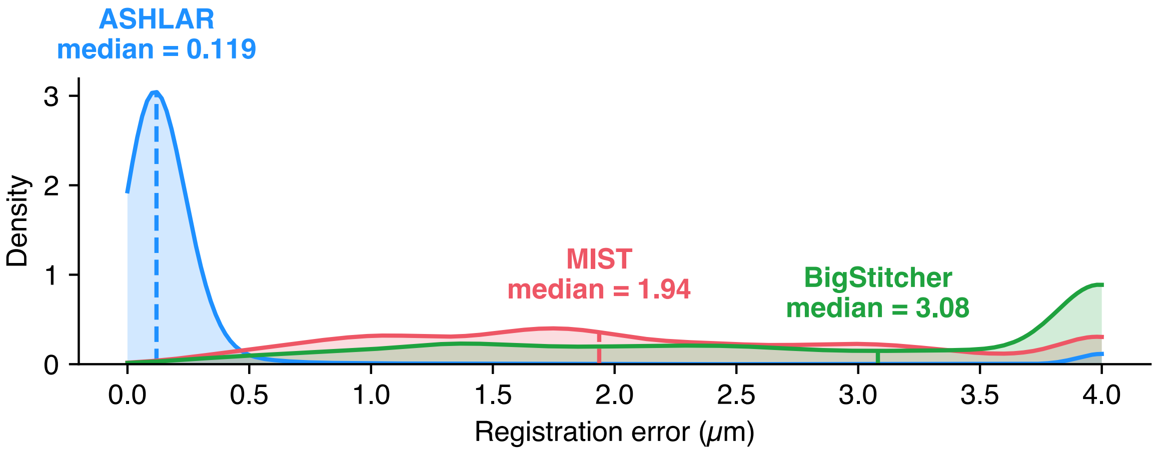

B ASHLAR error field

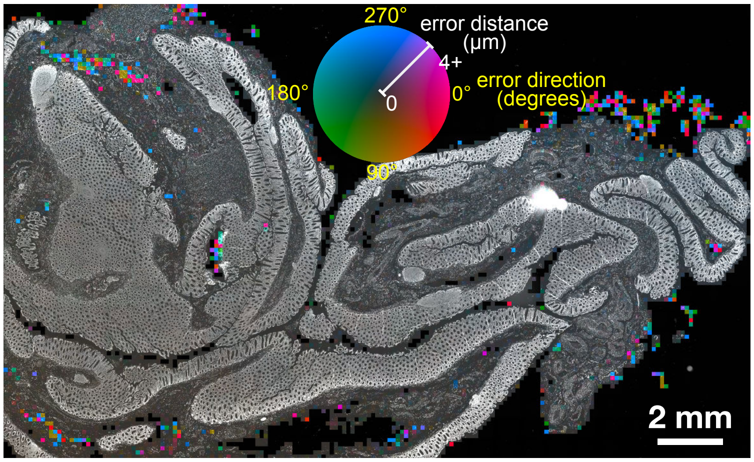

C MIST error field

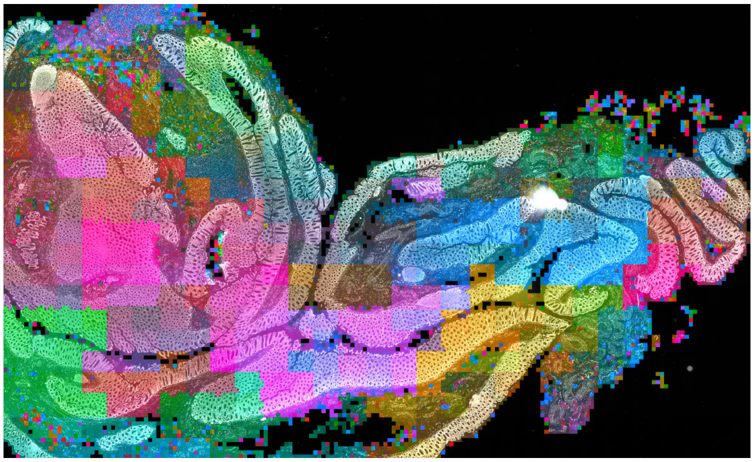

D BigStitcher error field

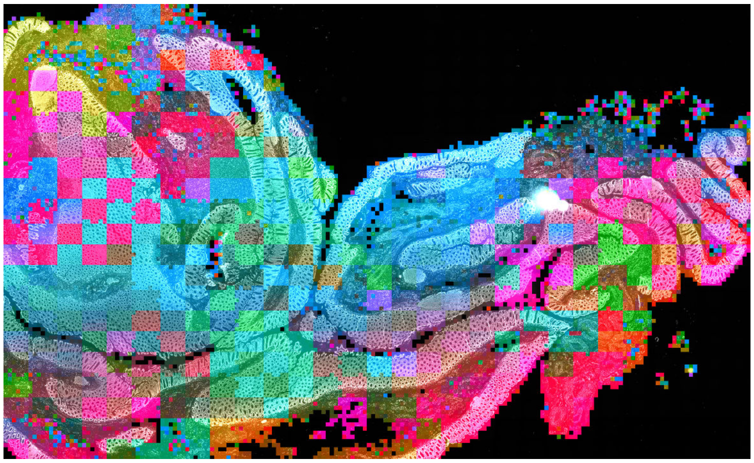

(A) Local registration error distance distributions for ASHLAR, MIST, and BigStitcher mosaic images of two t-CyCIF cycles of the human colon section described in the manuscript. Distances at the upper end in this plot as well as in panels B-D were clipped to 4 μm to highlight the relevant data. **(B-D)** Heatmaps of local registration error for the three tools. Direction (hue) and magnitude (intensity) at 200-pixel resolution are overlaid on the nuclear stain image.

Figure S2: Registration accuracy comparison on CODEX human tonsil

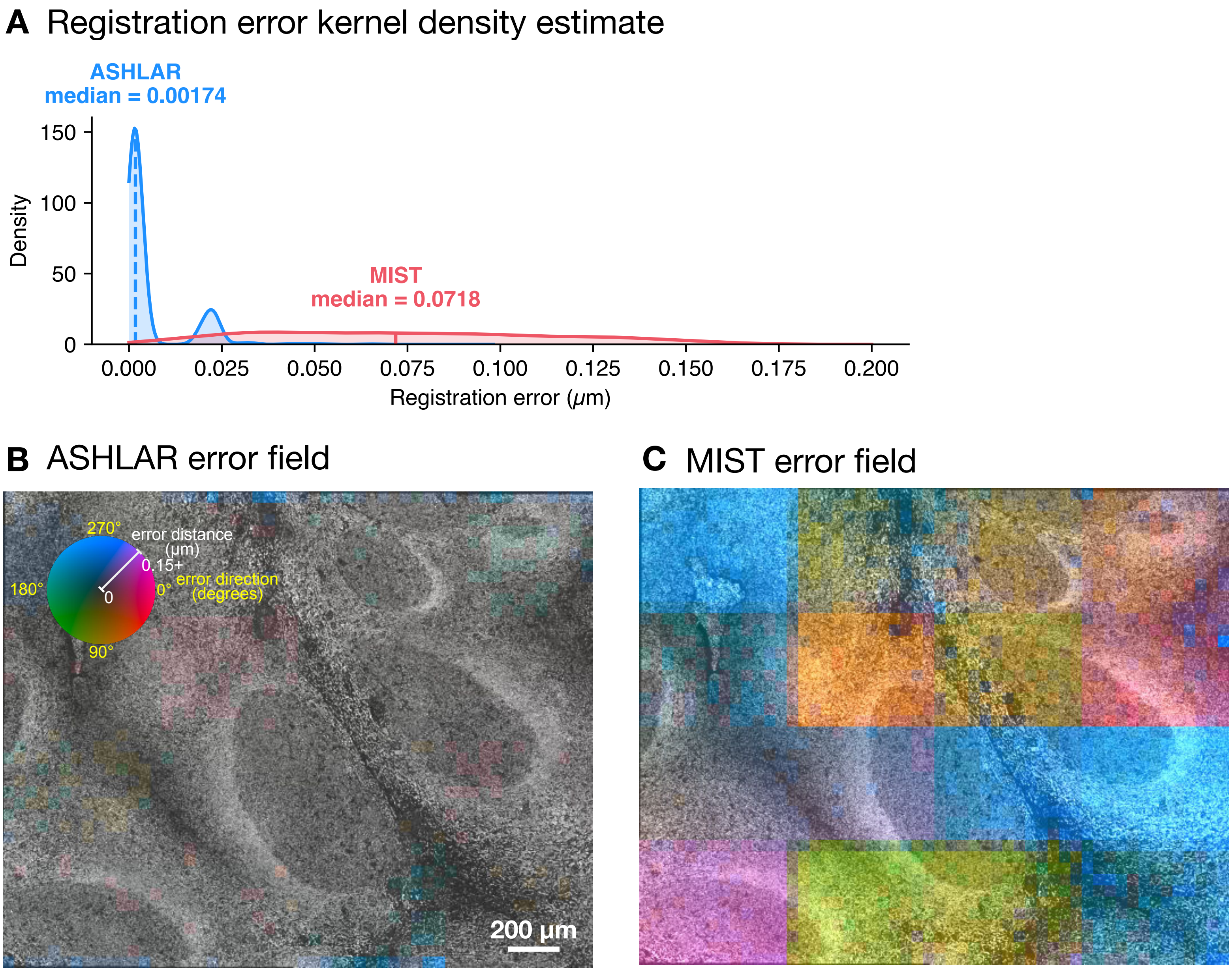

(A) Local registration error distance distributions for ASHLAR and MIST mosaic images of two CODEX cycles of a human tonsil section . Distances at the upper end in this plot as well as in panels B and C were clipped to 0.2 μm to highlight the relevant data. **(B,C)** Heatmaps of local registration error for the two tools. Direction (hue) and magnitude (intensity) at 200-pixel resolution are overlaid on the nuclear stain image.

Figure S3: Registration accuracy comparison on CODEX human spleen

A Registration error kernel density estimate

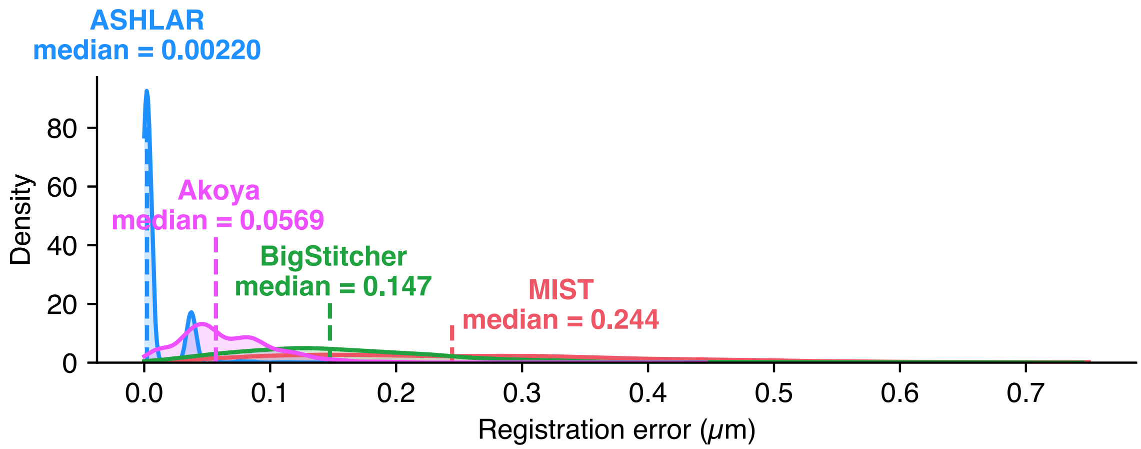

B ASHLAR error field

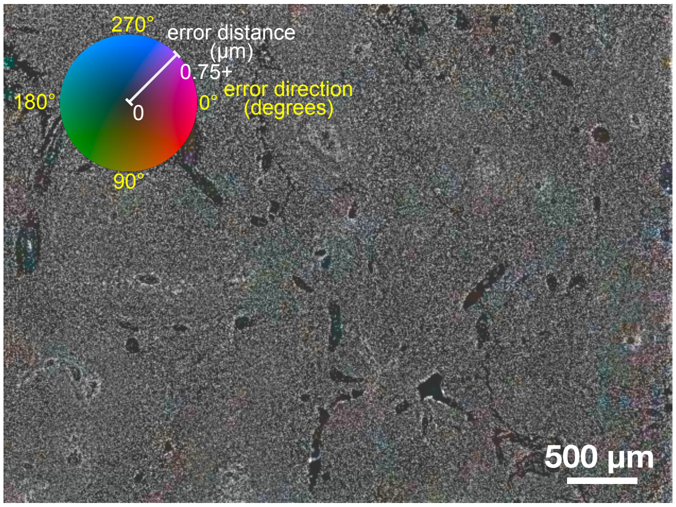

C MIST error field

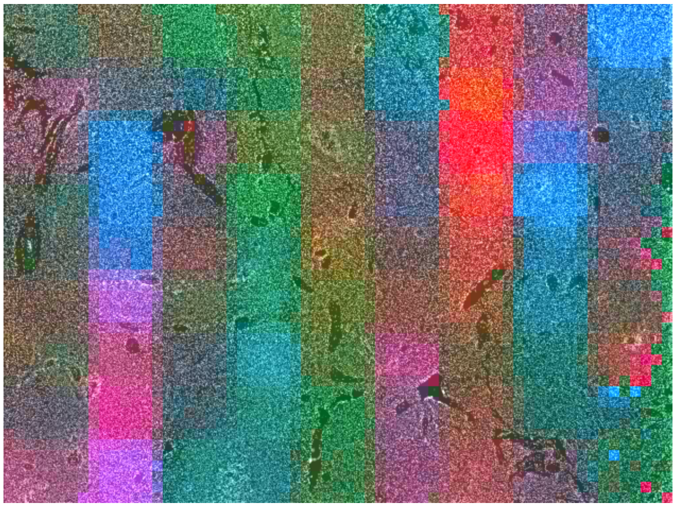

D BigStitcher error field

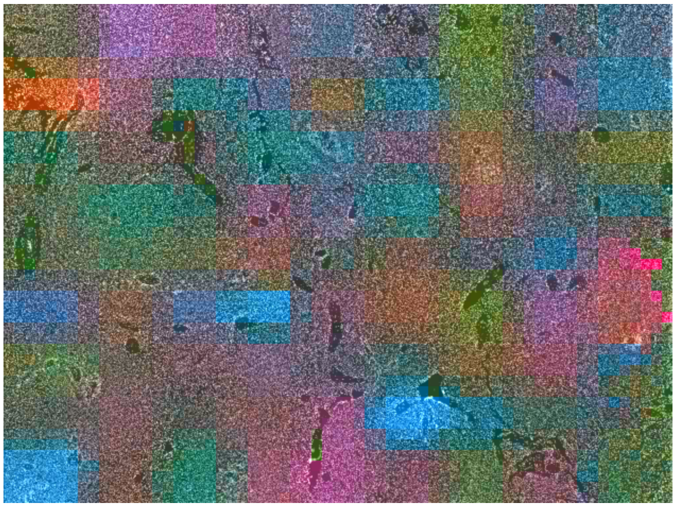

E Akoya CODEX pipeline error field

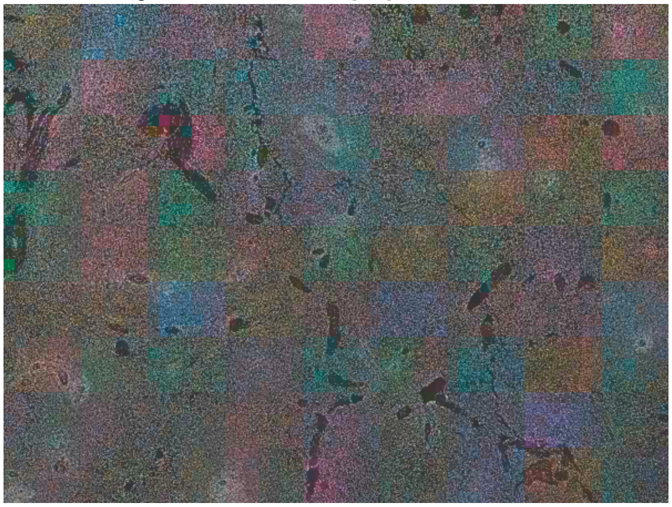

(A) Local registration error distance distributions for mosaic images from ASHLAR, MIST, BigStitcher, and the proprietary Akoya CODEX Processor of two CODEX cycles of a human spleen section. Distances at the upper end in this plot as well as in panels B-E were clipped to 0.75 μm to highlight the relevant data. **(B-E)** Heatmaps of local registration error for the four tools. Direction (hue) and magnitude (intensity) at 200-pixel resolution are overlaid on the nuclear stain image.

Figure S4: Registration accuracy comparison on CyCIF human TMA

A Registration error kernel density estimate

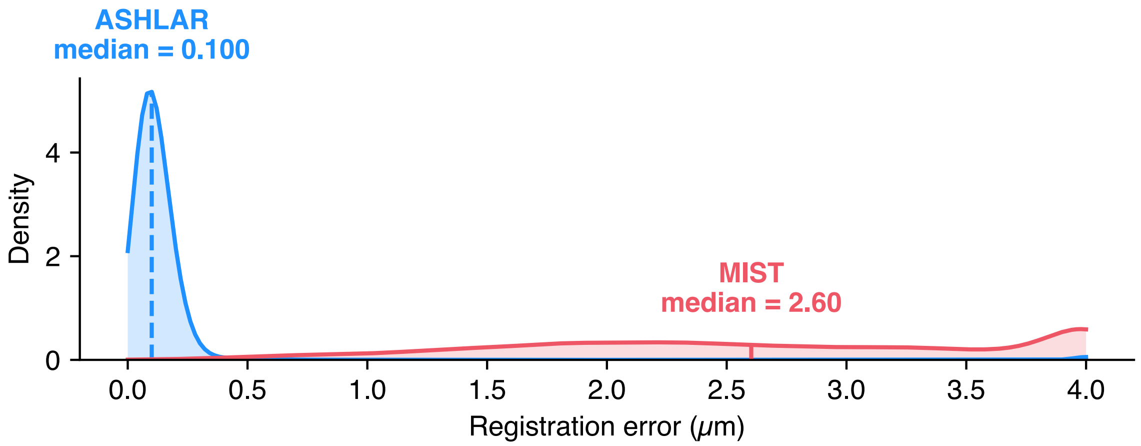

B ASHLAR error field

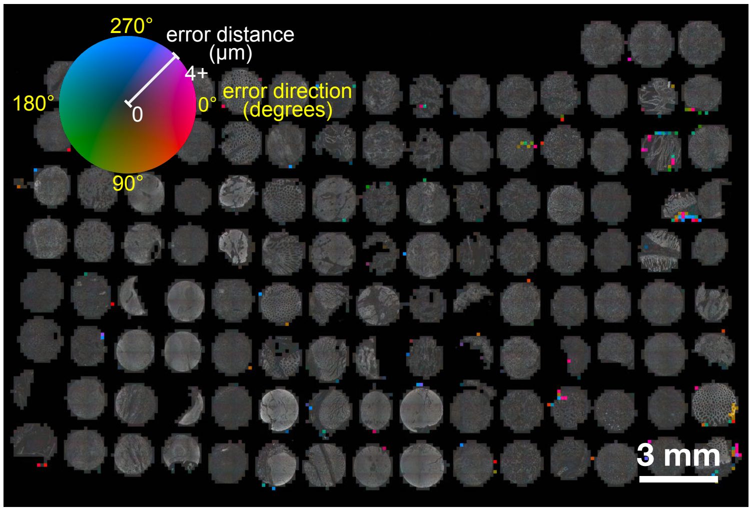

C MIST error field

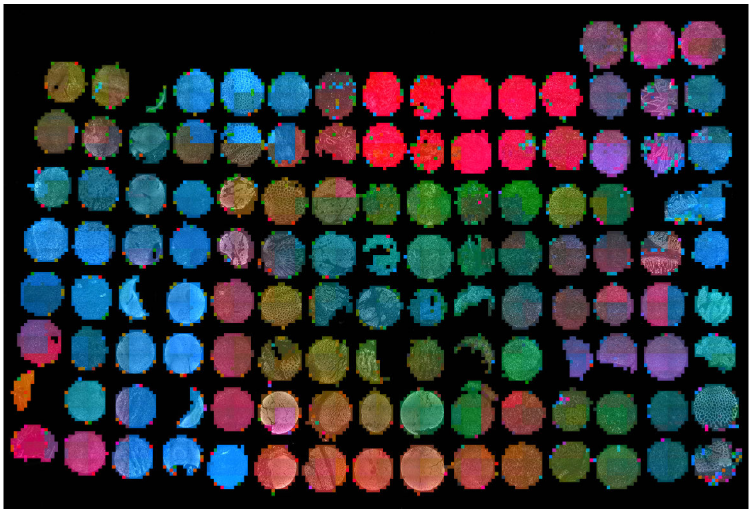

(A) Local registration error distance distributions for mosaic images from ASHLAR and MIST of two t-CyCIF cycles of a human tissue microarray (TMA). Distances at the upper end in this plot as well as in panels B and C were clipped to 4 μm to highlight the relevant data. **(B,C)** Heatmaps of local registration error for the two tools. Direction (hue) and magnitude (intensity) at 200-pixel resolution are overlaid on the nuclear stain image.

Figure S5: Registration accuracy comparison on CyCIF human tonsil

A Registration error kernel density estimate

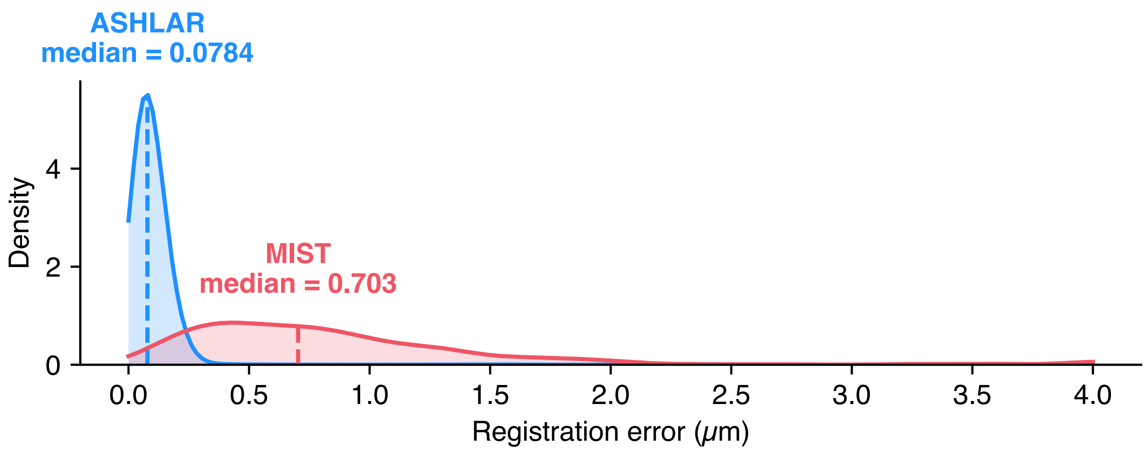

B ASHLAR error field

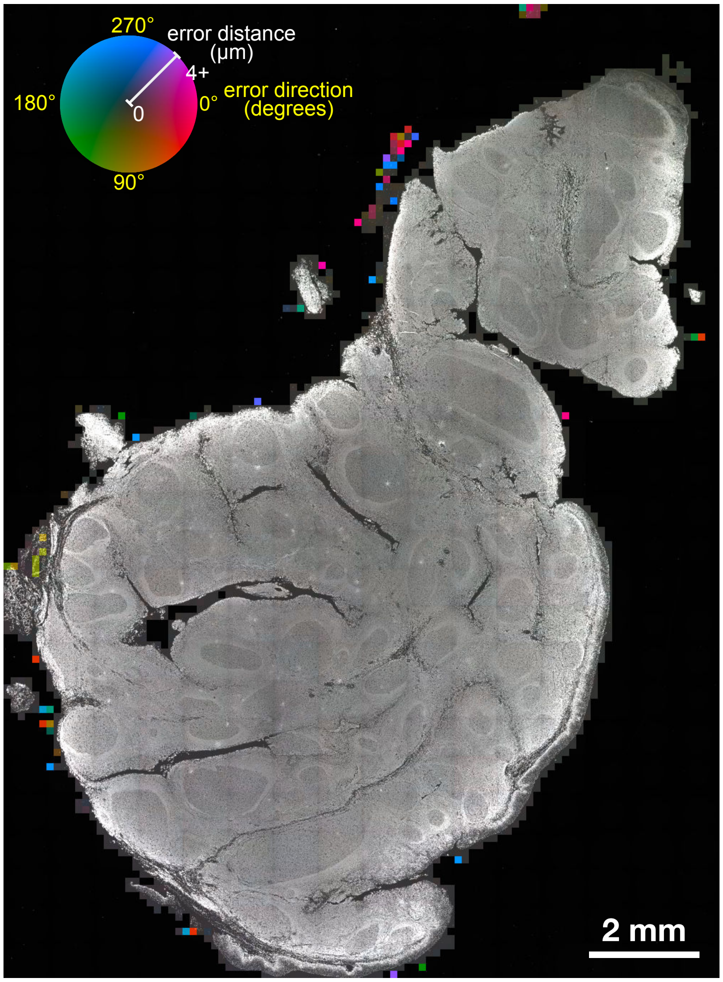

C MIST error field

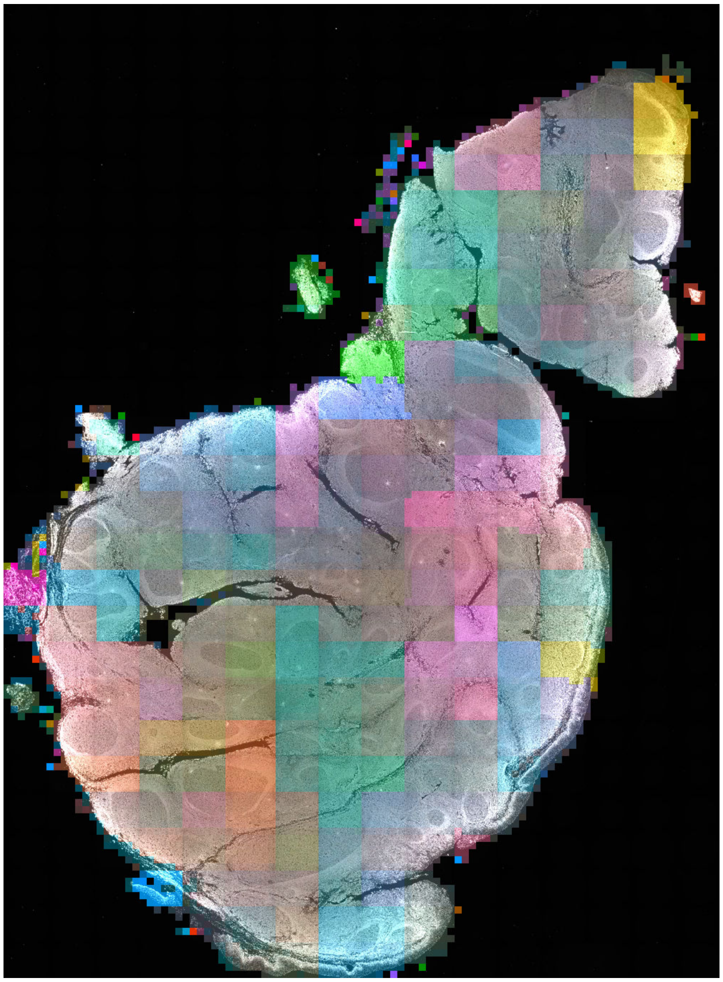

(A) Local registration error distance distributions for mosaic images from ASHLAR and MIST of two t-CyCIF cycles of a human tonsil section. Distances at the upper end in this plot as well as in panels B and C were clipped to 4 μm to highlight the relevant data. **(B,C)** Heatmaps of local registration error for the two tools. Direction (hue) and magnitude (intensity) at 200-pixel resolution are overlaid on the nuclear stain image.

Figure S6: Registration accuracy of Zeiss Zen software on cyclic IF rat brain

A Registration error kernel density estimate

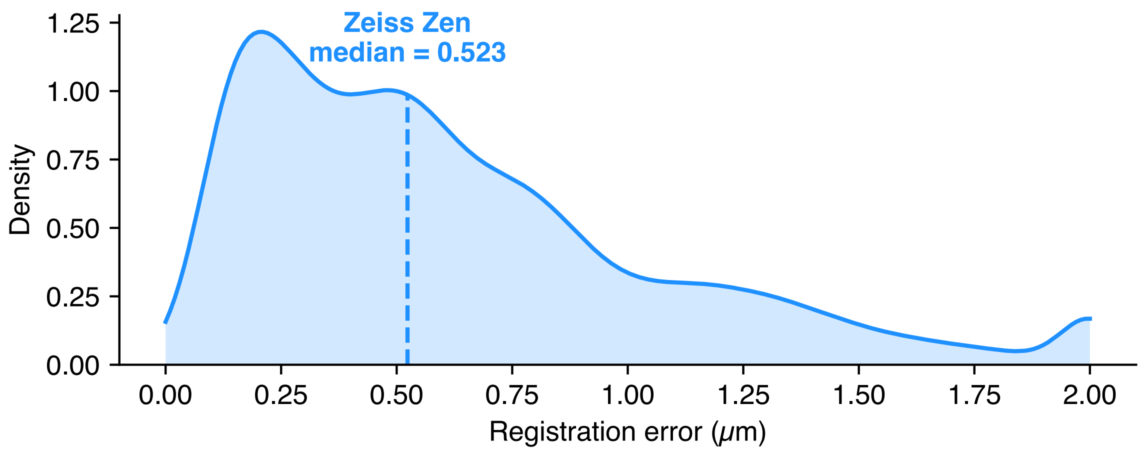

B Zeiss Zen error field

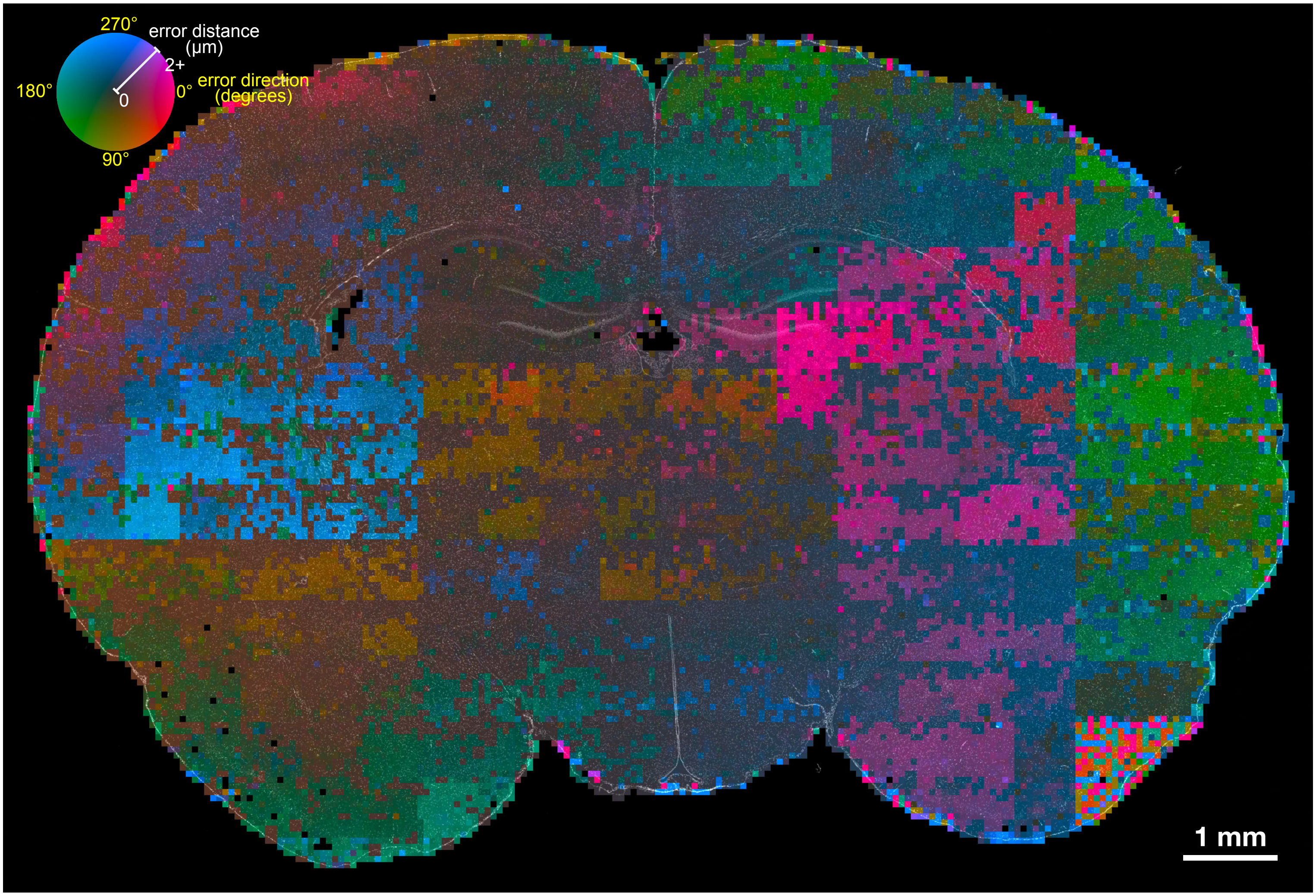

(A) Local registration error distance distributions for mosaic images from Zeiss Zen software of two cyclic IF cycles of a rat brain section. Distances at the upper end in this plot as well as in panel B were clipped to 2 μm to highlight the relevant data. **(B)** Heatmap of local registration error. Direction (hue) and magnitude (intensity) at 200-pixel resolution are overlaid on the nuclear stain image.

Figure S7: ASHLAR runtime vs. dataset size

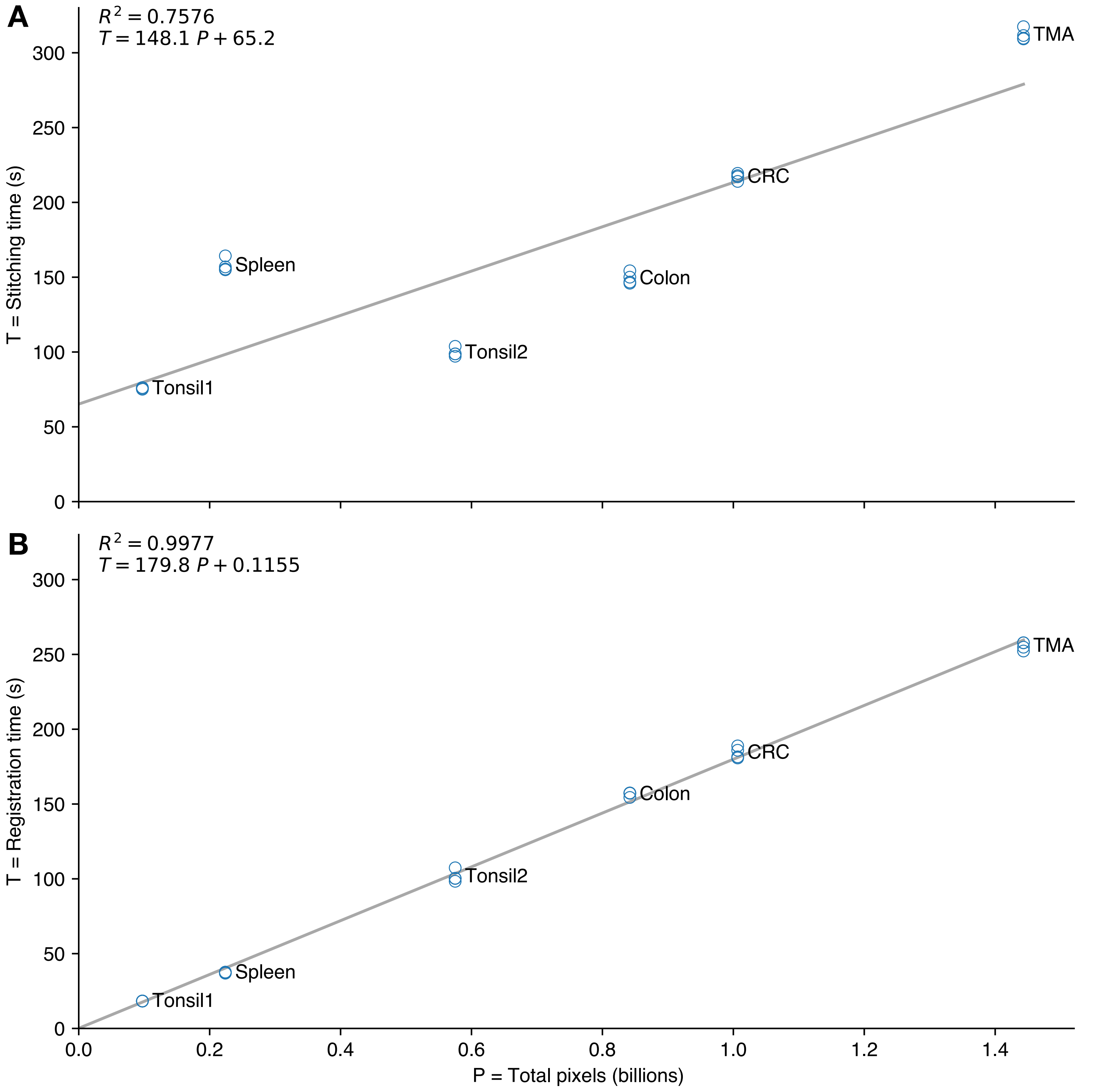

ASHLAR runtime for the stitching phase **(A)** and registration phase **(B)** vs. dataset size in total pixels per imaging cycle. Data points are shown for four runs of each of six datasets along with a linear fit. Datasets are summarized in **Supplementary Table 4**.

SUPPLEMENTARY TABLE 1. Published image data stitched and registered using ASHLAR.

Authors	Year	DOI	Tissue type	Tissue or organ of origin	WSI	TMA
Schapiro et al	2021	doi.org/10.1038/s41592-021-01308-y	Normal tonsil, colorectal cancer	Tonsil (2), colon (3), TMA (2)	5	2
Rashid et al	2021	doi.org/10.1038/s41551-021-00789-8	Lung carcinoma	Lung (1),	1	0
Duraiswamy et al	2021	doi.org/10.1016/j.ccell.2021.10.008	Ovarian cancer	Ovarian (19)	19	0
Krueger et al	2021	doi.org/10.1109/TVCG.2021.3114786	Colorectal cancer, lung adenocarcinoma	Colon (2), lung (1)	3	0
Gaglia et al	2022	doi.org/10.1101/2021.05.16.443704	Breast carcinoma, ovarian carcinoma	Breast (4), ovarian (0), TMA (6)	4	6
Liu et al	2021	doi.org/10.1038/s41591-021-01331-8	Melanoma	Skin (32)	32	0
Keenan et al	2021	dx.doi.org/10.1158%2F1078-0432.CCR-20-3089	Breast carcinoma	Breast (12)	12	0
Hemming et al	2021	dx.doi.org/10.1158%2F1078-0432.CCR-20-3538	Gastrointestinal stromal tumor	Gastrointestinal tract	0	1
Mehta et al	2021	doi.org/10.1038/s43018-020-00148-7	Breast carcinoma	Breast (16)	16	0
Iorgulescu et al	2021	doi.org/10.1158/1078-0432.CCR-20-2291	Glioblastoma	Brain	2	0
Ringel et al	2021	doi.org/10.1016/j.cell.2020.11.009	Colorectal tumors	Colon (14)	14	0
Chandrashekar et al	2020	doi.org/10.1126/science.abc4776	Lung	Lung (2)	2	0
Hemming et al	2020	doi.org/10.1200/po.19.00287	Sarcoma	Liver (2), sacrum (1)	3	0
Färkkilä et al	2020	doi.org/10.1038/s41467-020-15315-8	Ovarian cancer	Ovarian (19)	19	0
Gaglia et al	2020	doi.org/10.1038/s41556-019-0458-3	Colorectal tumors	Colon (1)	1	0
Krueger et al	2019	doi.org/10.1109/TVCG.2019.2934547	Melanoma, breast carcinoma, lung adenocarcinoma	Skin (1), breast (1), lung (1)	3	0
Du et al	2019	doi.org/10.1038/s41596-019-0206-y	Normal tonsil, lung carcinoma	Tonsil (1), lung (1), lymph node (1), brain (1)	4	0
Nirmal et al	2022	doi.org/10.1101/2021.05.23.445310	Melanoma	Skin (22)	22	0
Lin et al	2022	doi.org/10.1101/2021.03.31.437984	Colorectal cancer	Colon (75), TMA (2)	75	2
Kalocsay et al	2021	doi.org/10.1101/2020.10.14.339952	Lung	Lung (1)	1	0
**Total**			**12 tumor/tissue types**		**238**	**11**

SUPPLEMENTARY TABLE 2. Microscopes Tested with ASHLAR.

Instrument	Type	Datasets Collected[1]	Objective	Field of View	Nominal Resolution[2]
RareCyte CyteFinder	Slide scanner	Colon, TMA, Tonsil2	10X/0.3NA	1.66 x 1.40 mm	1.06 µm
			20X/0.75NA	0.83 x 0.7 mm	0.42 µm
			40X/0.6NA	0.42 x 0.35 mm	0.53 µm
RareCyte Orion	Slide scanner		20X/0.75NA	0.66 x 0.66 mm	0.42 µm
			40X/0.95NA	0.33 x 0.33 mm	0.33 µm
GE IN Cell Analyzer 6000	Slide scanning mode	CRC	10X/0.45NA	1.3 x 1.3 mm	0.70 µm
			20X/0.75NA	0.66 x 0.66 mm	0.42 µm
			40X/0.95NA	0.33 x 0.33 mm	0.33 µm
			60X/0.95NA	0.22 x 0.22 mm	0.33 µm
GE IN Cell Analyzer 6000	Confocal mode		60X/0.95NA	0.22 x 0.22 mm	0.21 µm
Zeiss Axio Scan.Z1	Slide scanner		10X/0.45NA	1.3 x 1.3 mm	0.70 µm
			20X/0.8NA	0.66 x 0.66 mm	0.40 µm
Zeiss Axio Observer.Z1	Slide scanner	Tonsil1	20X/0.8NA	0.83 x 0.66 mm	0.40 µm
Keyence BZ-X800	Slide scanner	Spleen	Keyence BZ-X800	0.72 x 0.54 mm	0.42 µm

[1] See Supplementary Table 4 for details on the listed datasets.

[2] The nominal resolution (r) was determined using the formulae: (r) = $0.61\lambda/NA$ for widefield or (r) = $0.4\lambda/NA$ for confocal microscopy (λ = 520 nm). The actual resolution depends on optical properties, the thickness of the tissue section, and both the alignment and the quality of the optical components used.

SUPPLEMENTARY TABLE 3. Antibodies used for CyCIF imaging of the human colon specimen.

Cycle Number	Channel Number	Target Name	RRID identifier	Fluorophore	Clone	Vendor	Catalog Number
1	1	DNA	AB_10626776	Hoechst 33342	-	Cell Signaling Technology	4082S
1	2	Rabbit-IgG	AB_2534114	Alexa Fluor 488	-	Invitrogen	A-11070
1	3	Rat-IgG	AB_2535855	Alexa Fluor 555	-	Invitrogen	A-21434
1	4	Mouse-IgG	AB_2535806	Alexa Fluor 647	-	Invitrogen	A-21237
2	5	DNA	AB_10626776	Hoechst 33342	-	Cell Signaling Technology	4082S
2	6	Na/K ATPase	AB_2798866	-	D4Y7E	Cell Signaling Technology	23565
2	7	CD3	AB_2889189	-	CD3-12	Abcam	ab11089
2	8	Cellular tumor antigen p53	AB_2206626	-	DO7	Dako	M7001
3	9	DNA	AB_10626776	Hoechst 33342	-	Cell Signaling Technology	4082S
3	10	Antigen Ki67	AB_2687824	Alexa Fluor 488	D3B5	Cell Signaling Technology	11882
3	11	Pan-cytokeratin	AB_11217482	eFluor 570	AE1/AE3	eBioscience/Thermo Fisher	41-9003-80
3	12	Aortic smooth muscle actin	AB_2574361	eFluor 660	1A4	eBioscience/Thermo Fisher	50-9760-80
4	13	DNA	AB_10626776	Hoechst 33342	-	Cell Signaling Technology	4082S
4	14	CD8a	AB_2574412	Alexa Fluor 488	AMC908	eBioscience/Thermo Fisher	53-0008-80
4	15	CD4	AB_2573601	eFluor 570	N1UG0	eBioscience/Thermo Fisher	41-2444-80
4	16	CD45	AB_493034	Alexa Fluor 647	HI30	BioLegend	304020
5	17	DNA	AB_10626776	Hoechst 33342	-	Cell Signaling Technology	4082S
5	18	CD45RO	AB_528823	Alexa Fluor 488	UCHL1	BioLegend	304212
5	19	CD11c		Alexa Fluor 555	D3V1E	Cell Signaling Technology	77882BC
5	20	PD-L1	AB_2728832	Alexa Fluor 647	E1L3N	Cell Signaling Technology	15005
6	21	DNA	AB_10626776	Hoechst 33342	-	Cell Signaling Technology	4082S
6	22	CD68	AB_2798886	Alexa Fluor 488	D4B9C	Cell Signaling Technology	24850
6	23	FoxP3	AB_2573608	eFluor 570	236A/E7	eBioscience/Thermo Fisher	41-4777-80
6	24	PD-1	AB_2728811	Alexa Fluor 647	EPR4877(2)	Abcam	ab201825
7	25	DNA	AB_10626776	Hoechst 33342	-	Cell Signaling Technology	4082S
7	26	CD20	AB_10734358	Alexa Fluor 488	L26	eBioscience/Thermo Fisher	53-0202-82
7	27	Phospho-histone H3.1 (Ser10)	AB_10694639	Alexa Fluor 555	D2C8	Cell Signaling Technology	3475S
7	28	CD31	AB_2857973	Alexa Fluor 647	EPR3094	Abcam	ab218582
8	29	DNA	AB_10626776	Hoechst 33342	-	Cell Signaling Technology	4082S
8	30	E-cadherin	AB_10691457	Alexa Fluor 488	24E10	Cell Signaling Technology	3199
8	31	Vimentin	AB_10859896	Alexa Fluor 555	D21H3	Cell Signaling Technology	9855
8	32	Catenin beta-1	AB_10691326	Alexa Fluor 647	L54E2	Cell Signaling Technology	4627
9	33	DNA	AB_10626776	Hoechst 33342	-	Cell Signaling Technology	4082S
9	34	CD163	AB_2889155	Alexa Fluor 488	EPR14643-36	Abcam	ab218293
9	35	Histone H3.1	AB_2799990	phycoerythrin	D1H2	Cell Signaling Technology	82241
9	36	Phospho-Histone H2AX (Ser139)	AB_2114994	Alexa Fluor 647	2F3	BioLegend	613407

SUPPLEMENTARY TABLE 4. Description of datasets analyzed.

Name	Figures	Tile Dimensions	Grid Dimensions	Pixel Size (μm)	Method	Instrument	Tissue	Source	Location
Colon	4,5,S1	1280 x 1080	29 x 21	0.65	t-CyCIF	RareCyte Cytefinder	human colon	Published as part of this work	https://dx.doi.org/10.7303/syn25826362
CRC	6B	2048 x 2048	15 x 16	0.325	H&E brightfield	GE INCell 6000	human colorectal adenocarcinoma	Unpublished	
Tonsil1	S2	2752 x 2208	4 x 4	0.227	CODEX	Zeiss Axio Observer Z1	human tonsil	Synapse (MCMICRO exemplar data)	https://www.synapse.org/#!Synapse:syn24849819
Spleen	S3	1920 x 1440	9 x 9	0.38	CODEX	Keyence BZ-X800	human spleen	HuBMAP (University of Florida TMC / J. P. Aponte)	https://dx.doi.org/10.35079/HBM355.JDLK.244
TMA	S4	1280 x 1080	36 x 29	0.65	t-CyCIF	RareCyte Cytefinder	human, various	Synapse (EMIT exemplar data)	https://www.synapse.org/#!Synapse:syn22345748/wiki/609239
Tonsil2	S5	1280 x 1080	15 x 25	0.65	t-CyCIF	RareCyte Cytefinder	human tonsil	Unpublished	
Brain2	S6	2048 x 2048	22 x 15 (approximate)	0.325	cyclic IF	Zeiss Axio Imager Z2	rat brain	Figshare (Maric et al.)	https://doi.org/10.6084/m9.figshare.13731585.v1
